# Equity in the distribution of general practitioners and specialists in Iran: a health needs-adjusted analysis using the Robin Hood index (2006–2019)

**DOI:** 10.1186/s12960-026-01057-z

**Published:** 2026-04-28

**Authors:** Nader Jahanmehr, Soheila Damiri, Zahra Meshkani

**Affiliations:** 1 Department of Health Policy and Management, School of Public Health and Safety, Shahid Beheshti University of Medical Sciences, Tehran, Iran; 2 Department of Health Management, Policy, and Economics, School of Public Health, Tehran University of Medical Sciences, Tehran, Iran; 3 Department of Health Economics, School of Health Management and Information Sciences, Iran University of Medical Sciences, Tehran, Iran; 4 National Center for Health Insurance Research, Tehran, Iran

**Keywords:** Human resource, Health equity, Needs assessment, Resource allocation, Robin Hood index

## Abstract

**Backgrounds:**

Physicians are among the most vital healthcare resources. The equitable distribution of human resources could help policymakers to reach equity as the most important health care goal. In Iran, addressing the shortage of qualified healthcare personnel requires policymakers to consider the population’s health needs. The present study evaluated the equity in the distribution of general practitioners (GPs) and specialists (SPs) in Iran, both before and after adjusting for health needs. Disability-adjusted life years (DALYs) and mortality were used as indicators of health needs.

**Method:**

This study is based on a retrospective cross-sectional design and looks at the distribution of GPs and SPs across Iranian provinces over 14 years (2006–2019) by the Robin Hood index. Data on the number of GPs and specialists, as well as provincial mortality, were obtained from the Statistical Center of Iran. DALYs were sourced from the Global Burden of Disease (GBD) study. Additionally, Lorenz curves were plotted to visualize the distribution by use of Excel 2016. The R.4.5.1 software (package EconGeo) was used to analysing the data.

**Results:**

During the study period, the Robin Hood index for GPs, based on population size, mortality, and DALYs, was 0.104, 0.127, and 0.111, respectively; for SPs, the corresponding values were 0.118, 0.133, and 0.124. The greatest equity in the distribution of GPs and SPs was observed in 2016 and 2019, respectively. Overall, less than 13% of the physicians should be redistributed to reach equity. Tehran exhibited both the lowest disparity in SP distribution and the highest disparity in GP distribution, relative to mortality and DALYs.

**Conclusion:**

Overall, the distribution of GPs and SPs in Iran was close to the equity line; however, SP distribution was closer to the equity line than that of GPs. The distribution appeared more equitable when evaluated using population size, DALYs, and mortality, respectively. Using population size alone as a planning tool can be misleading. Instead, health workforce policies should take a broader view—one that includes how people use services, how demographics are changing, and how local socioeconomic conditions shape health needs. Effective strategies—including financial incentives, career pathway development, implementation of post-graduation “return-to-service” programs, adoption of telemedicine, and integration of artificial intelligence—can support physician retention and, in turn, foster a more equitable distribution of medical professionals.

## Introduction

The World Health Organization (WHO) has long recognized access to healthcare as a fundamental human right and emphasized the importance of fair, equitable, and efficient allocation of healthcare resources to uphold this principle. Achieving health equity is both a moral imperative and a practical necessity for health systems worldwide [1]. In healthcare, equity is defined by the interventions patients receive, which should be based on their needs [2]. Although the importance of equity for the efficiency of the health care system, persistent disparities in healthcare access, utilization, and outcomes continue to compromise progress toward universal health coverage (UHC) [3]. There is growing recognition that the progressive realization of UHC relies on a sufficient, equitably distributed, and high-performing health workforce [4]. Human resources are not only the largest cost component in most healthcare systems, but also they are the most important vital input, which is critical for healthcare development [5, 6]. Based on Mirahmadizadeh et al. the concentration of physicians and other qualified health professionals contributes to a reduction in mortality rates [7]. These human resources clearly influence other health indicators and play a key role in improving healthcare efficiency and quality of life.

The WHO 2016 Global Strategy on Human Resources for Health seeks to support Sustainable Development Goal (SDG) 3c, to “increase health financing and the recruitment, training, and retention of the health workforce” [8]. Estimates indicate substantial global variation in the availability of human resources for health. For instance, according to data from the GBD Collaborative Network based on SDG monitoring indicators, SDG Indicator 3.c.1—the number of physicians, nurses and midwives, and pharmacists per 1000 population—ranged from 0.471 in the Central African Republic to 27.44 in Norway in 2017 [8]. According to the GBD 2019 Human Resources for Health Collaborators, the global health workforce in 2019 was estimated at 104 million people, including 12.8 million physicians, 29.8 million nurses and midwives, 4.6 million dental personnel, and 5.2 million pharmaceutical personnel. Yet these numbers fall short of what is needed: at least 20.7 physicians, 70.6 nurses/midwives, 8.2 dentistry personnel, and 9.4 pharmaceutical personnel per 10,000 population are required to achieve a UHC effective coverage index of 80. The global shortfall in 2019 was staggering—6.4 million physicians, 30.6 million nurses and midwives, 3.3 million dentists, and 2.9 million pharmacists [6]. Human resources for health (HRH) reform has gained global momentum; the workforce crisis has been identified by international consensus, and taskforces are implementing by WHO recommended interventions: educational reforms, regulation, financial incentives, and personal/professional support for health workers are acting to increase supply, enhance distribution and incentivize retention of health workers in neglected areas. Impressive gains have been made, but to achieve the SDGs, continued effort is needed to develop evidence-based human resources for health policy [8]. Policymakers must understand how healthcare resources are distributed in relation to population health needs. To quantify these needs, the health needs index (HNI) was developed. Key determinants of health needs include disease prevalence, health indicators such as mortality rates, socioeconomic conditions, healthcare infrastructure, and demographic characteristics [9].

Iran is no exception to this global pattern. In 2019, Iran had 12.2 physicians, 27.1 nurses and midwives, 4.1 dentists, and 7.5 pharmacists per 10,000 people, falling short of the minimum required thresholds to reach UHC coverage targets. Achieving a UHC index of 80 would require the addition of 11.7, 41.7, 3.3, and 6.7 professionals per 10,000 population, respectively—translating into a national shortage of approximately 98,470 physicians, 351,394 nurses/midwives, 27,573 dentists, and 56,300 pharmacists [6]. Numerous studies published in recent years have examined disparities in health risk factors [10–13] and health outcomes at the provincial level [14, 15]. These differences reflect regional disparities in health needs and highlight the necessity of regional planning and need-based allocation of health resources to enhance the efficiency of health resource utilization in Iran. In recent years, numerous studies have been conducted to assess equity in the distribution of human health resources, particularly physicians in Iran [16–23]. Most of these studies have primarily employed measurement tools such as the Gini coefficient [16–19], Lorenz curve [17–19], and Atkinson index [20], with calculations mainly based on population size in each region, while giving limited attention to health needs. One of the key challenges in health systems is how to allocate resources equitably in line with the varying health needs of populations. Need-based allocation models aim to distribute resources by incorporating indicators such as age, sex, health status, and socioeconomic conditions. Despite advances in methodology, choosing appropriate indicators and ensuring reliable data remain persistent challenges in applying these models across regions [24]. One of the key indicators proposed for need-based resource allocation in health systems is the DALY. Carla Castillo-Laborde identified the relationship between the density of health workers and the burden of disease (measured in DALYs) as follows: “An increase of one unit in the density of health workers per 1000 population is associated with an average reduction in the total burden of disease by 1% to 3%.” [25]. Allocating health professionals based on population size alone fails to capture these nuanced needs. Instead, needs-based allocation, which adjusts for local disease burden and mortality, offers a more equitable and effective approach. While many global studies have employed various inequality indices such as the Gini coefficient, Theil index, and concentration index, [16–20] relatively few have focused on physician distribution adjusted for DALYs—particularly in the Iranian context. The present study aims to assess the equity of the distribution of GPs and SPs across Iran using a health needs–adjusted framework. The Gini index is a commonly used measure for illustrating the equity of a resource, such as income or other economic assets. However, policymakers often require more detailed insights to achieve equitable distribution. In this regard, the Robin Hood index serves as a valuable tool, as it indicates the proportion of human resources that must be redistributed to achieve equity [26–28]. The present study apply the Robin Hood index in combination with health need indicators—including DALYs and mortality—and compare the results with those derived from a traditional population-based approach to help policymakers for redistributed the health human resources based on health needs.

## Method

### Study design and data sources

This study is based on a retrospective cross-sectional design and looks at the distribution of GPs and SPs across Iranian provinces over 14 years (2006–2019). Our goal was to examine how equitable this distribution was, taking into account varying health needs in different provinces. The data were gathered from two main sources. The Statistical Center of Iran provided data on provincial number of populations, mortality cases, GPs and SPs. For data on the burden of disease, we used DALY estimates from the GBD study 2021 [29]. Although data for the years 2020 and 2021 were available, we excluded these years from the final analysis due to the impact of the COVID-19 pandemic. The outbreak placed a highly uneven burden of disease across different regions of the country, which could have significantly distorted the underlying trends in physician distribution and health needs. Given the sharp decline in disease burden in the years following the pandemic, we concluded that including these years might lead to misleading interpretations of long-term patterns. Therefore, we limited our study to the pre-pandemic period (2006–2019) to preserve the consistency and interpretability of the results. Although this study relied solely on publicly available, non-identifiable data and did not involve direct interaction with human subjects, it was approved by the Research Ethics Committee of Shahid Beheshti University of medical sciences, under the ethics code IR.SBMU.SME.REC.1401.020.

### Equity assessment

**Fig. 1:**
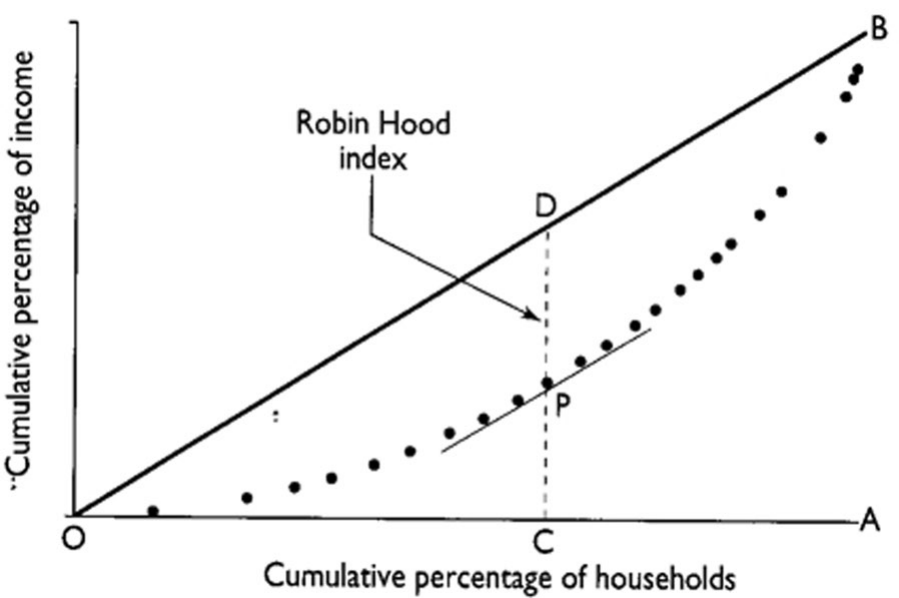
Robin Hood index

We opted for the Robin Hood index over the more traditional Gini coefficient because of its clear interpretability in health resource allocation. The index reflects the proportion of total resources that would have to be redistributed to achieve equal distribution. It is represented by the maximum vertical distance between the Lorenz Curve and the 45-degree line of perfect equality [30] (illustrated in Fig. 1).

To evaluate equity in the distribution of GPs and SPs across Iranian provinces, we calculated the Robin Hood index under three different need-based scenarios: population size, crude mortality, and disability-adjusted life years (DALYs).

In each scenario, provinces were first sorted in ascending order based on their physician-to-need ratio (e.g., physicians per capita, per death, or DALY). This ordering ensured that areas with the lowest levels of physician availability relative to need appeared first in the analysis. Following this, cumulative shares were calculated for each province, both for the selected need indicator and for the number of physicians. These cumulative percentages formed the basis for constructing Lorenz curves, which visually represented the degree of inequality in physician distribution under each scenario. The Robin Hood index (H) was then calculated as the maximum absolute difference between the cumulative share of need and the cumulative share of physicians. Mathematically, it is expressed as:

$$ H\, = \,Max\,\left\lceil {\frac{{E_{{_{i} }} }}{{E_{t} }} - \,\frac{{A_{i} }}{{A_{t} }}} \right\rceil \,\,{\mathrm{Number}}\,{\mathrm{of}}\,{\mathrm{physicians}}\,{\mathrm{in}}\,{\mathrm{province}}\,{\mathrm{i}}\, $$

$$ E_{i} \,:\,{\mathrm{cumulative}}\,{\mathrm{share}}\,{\mathrm{of}}\,{\mathrm{physicians}}\,{\mathrm{in}}\,{\mathrm{province}}\,{\mathrm{i}} $$

$$ E_{t} \,:\,\,{\mathrm{total}}\,{\mathrm{number}}\,{\mathrm{of}}\,{\mathrm{physicians}}\,\left( {{\mathrm{also}}\,{\mathrm{equals}}\,{1}\,{\mathrm{in}}\,{\mathrm{cumulative}}\,{\mathrm{share}}\,{\mathrm{form}}} \right) $$

$$ A_{{i{\kern 1pt} }} \,:\,{\mathrm{cumulative}}\,{\mathrm{share}}\,{\mathrm{of}}\,{\mathrm{population}}\,{\mathrm{or}}\,{\mathrm{mortality}}\,{\mathrm{or,}}\,{\mathrm{DALYs}}\,{\mathrm{in}}\,{\mathrm{province}} $$

$$ A_{t} \,:\,\,{\mathrm{total}}\,{\mathrm{need}}\,\left( {{\mathrm{which}}\,{\mathrm{equals}}\,{1,}\,{\mathrm{as}}\,{\mathrm{shares}}\,{\mathrm{are}}\,{\mathrm{normalized}}} \right) $$

The index ranges from 0 (perfect equity) to 1 (complete inequality), and it reflects the proportion of physician resources that would need to be reallocated to achieve an equal distribution. To translate the index into actionable terms, the number of physicians required to be redistributed was estimated by multiplying the Robin Hood index by the total number of physicians nationally:

$$ Redistributed\,physicians = \,E_{t} \, \times \,H $$

We further quantified the surplus or shortage of physicians in each province relative to an equitable distribution. This was done by calculating the expected number of physicians for each province based on its share of the national need and comparing it with the actual number of physicians available:

$$ Expected_{i} \, = \,Gap\, \times \,\left( {\frac{{A_{i} }}{{A_{t} }}} \right)\,\,\,\,Expected_{i} \, - \,Actual_{i} \, = \,_{i} E_{t} $$

Positive values in this redistribution gap indicate a surplus of physicians, while negative values indicate a shortage. These values provide an estimate of how physician resources would need to be shifted across provinces to achieve greater equity. Lorenz curves were plotted by use of Excel 2016. The Robin Hood index was calculated using the R 4.5.1 software with the EconGeo package. The corresponding command is provided in Appendix 1.

## Results

To assess equity in the geographic distribution of GPs and SPs in Iran, the Robin Hood index was calculated for each year between 2006 and 2019. The index was estimated based on population size, and then recalculated after adjusting for health needs using mortality and DALYs as need indicators.

Table 1 presents the number of GPs, SPs, mortality, and DALYs per 10,000 people in each Iranian province during the study period. During the study period, the number of GPs and SPs in Iran increased by approximately 21 and 27%, respectively, rising from 419 to 400 per 10,000 people in 2006 to 549 and 548 in 2019. Over the same period, the crude mortality rate slightly declined from 1.34 to 1.27 per 10,000 population, while the national average DALY (rate) increased marginally from 124 to 127.

**Table 1:** Provincial-level health indicators in Iran per 10,000 population (2006 and 2019)

Province	Population(*N*)		GPs		SPs		Mortality		DALY	
			Per 10,000 people							
	2006	2019	2006	2019	2006	2019	2006	2019	2006	2019
Ardebil	1,228,155	1,297,000	1.29	2.38	0.94	1.43	51.13	54.27	2704.89	2569.24
Isfahan	4,559,256	5,292,000	2.12	2.12	1.86	2.13	49.10	47.87	2490.40	2372.48
Alborz		2,865,000		1.62		1.83		39.77		2285.90
Ilam	545,787	597,000	2.20	3.18	1.34	2.78	44.19	42.70	2542.85	2475.95
East Azerbaijan	3,603,456	4,018,000	2.58	2.39	2.54	1.74	59.55	57.33	2887.85	2566.42
West Azerbaijan	2,873,459	3,398,000	1.83	1.78	1.25	1.76	51.95	45.92	2908.95	2457.57
Bushehr	886,267	1,230,000	2.93	2.08	1.47	2.15	59.15	36.45	2466.68	2105.19
Tehran	13,422,366	13,807,000	0.90	0.62	2.12	2.00	44.00	43.97	1821.08	2231.72
ChaharM and Bakhtiari	857,910	979,000	3.50	3.54	1.74	2.16	57.70	41.89	2340.81	2121.04
South Khorasan	636,420	809,000	1.40	2.56	6.51	3.08	63.98	50.25	2885.05	2364.21
Khorasan-e-Razavi	5,593,079	6,768,000	1.32	1.74	2.07	1.65	56.15	46.48	3157.51	2436.81
North Khorasan	811,572	892,000	2.11	2.07	1.06	2.23	57.15	54.92	2962.48	2454.72
Khuzestan	4,274,979	4,885,000	1.96	2.20	1.85	2.24	83.23	43.75	2774.28	2455.76
Zanjan	964,601	1,095,000	2.61	2.37	1.78	2.17	56.44	49.46	2805.74	2307.96
Semnan	589,742	750,000	3.34	2.25	3.22	2.41	48.51	48.41	2608.67	2328.04
Sistan and Baluchistan	2,405,742	2,978,000	1.54	1.86	0.95	1.67	66.20	43.25	3214.85	2495.33
Fars	4,336,878	5,006,000	1.82	2.37	1.50	2.59	58.99	44.38	2985.79	2640.94
Qazvin	1,143,200	1,322,000	2.06	2.13	1.03	1.75	48.67	47.53	2583.99	2324.89
Qom	1,046,737	1,373,000	1.14	1.00	1.36	1.36	52.44	49.70	2612.54	2064.62
Kurdistan	1,440,156	1,658,000	2.26	3.18	1.50	2.64	50.56	45.39	2883.04	2418.44
Kerman	2,652,413	3,299,000	2.00	2.37	1.24	2.19	40.31	42.54	2848.95	2465.70
Kermanshah	1,879,385	1,989,000	2.25	2.72	1.29	2.03	67.77	57.75	3163.54	2739.09
Kohgiluyeh and Boyer-Ahmad	634,299	744,000	1.97	3.86	1.25	2.88	96.47	38.60	2638.50	2305.47
Golestan	1,610,787	1,951,000	2.33	2.74	1.98	2.79	48.62	50.67	2998.59	2666.34
Gilan	2,404,861	2,562,000	2.74	2.51	1.85	2.11	61.71	71.71	2769.09	2735.01
Lorestan	1,716,527	1,793,000	1.92	2.30	1.30	1.99	54.14	51.34	2518.61	2296.35
Mazandaran	2,922,432	3,365,000	2.61	3.00	2.11	1.94	50.78	53.79	2577.67	2607.41
Markazi	1,351,257	1,467,000	2.07	2.25	1.30	2.51	58.21	54.50	2866.22	2462.02
Hormozgan	1,403,674	1,902,000	2.09	2.43	1.07	1.43	201.38	34.36	2676.85	2233.08
Hamadan	1,703,267	1,771,000	1.98	2.43	1.43	2.06	64.05	61.50	2996.21	2671.97
Yazd	990,818	1,213,000	2.94	2.89	2.19	2.08	46.60	42.28	2707.74	2161.46
Average	2,349,649	2,679,839	2.127	2.35	1.77	2.12	61.64	48.15	2746.65	2413.58
Min	545,787	597,000	0.9	0.62	0.94	1.36	40.31	34.36	1821.08	2064.62
Max	13,422,366	13,807,000	3.5	3.86	6.51	3.08	201.38	71.71	3214.85	2739.09

^*^Alborz was added as a province of Iran after 2006

The Robin Hood index and the number of redistributions required for GPs from 2006 to 2019 are presented in Table 2. On average, the Robin Hood index for GPs based on population size was 0.104, indicating a distribution close to equity. On average, 10% of GPs needed to be redistributed to achieve equitable distribution based on population size. When adjusted for health needs, the average Robin Hood index for GPs was 0.127 based on mortality and 0.111 based on DALY, suggesting that mortality contributed most to inequity in GP distribution. According to the annual trends, the most equitable distribution of GPs occurred in 2016 under all scenarios, whereas the highest inequality was observed in 2009, particularly when adjusted by DALYs.

**Table 2:** Robin Hood index and estimated number of GPs to be redistributed across provinces (2006–2019)

Year	Based on population size		Based on mortality		Based on DALYs	
	Robin Hood index	Physicians to be reallocated(*N*)	Robin Hood index	Physicians to be reallocated(*N*)	Robin Hood index	Physicians to be reallocated(*N*)
2006	0.106	1378	0.155	2015	0.121	1573
2007	0.099	1400	0.135	1909	0.112	1584
2008	0.118	1687	0.162	2316	0.121	1730
2009	0.124	1876	0.147	2223	0.133	2012
2010	0.119	1772	0.137	2040	0.128	1906
2011	0.109	1706	0.118	1847	0.111	1737
2012	0.093	1387	0.107	1596	0.102	1522
2013	0.106	1605	0.128	1938	0.116	1756
2014	0.103	1588	0.123	1897	0.110	1696
2015	0.100	1588	0.105	1667	0.107	1699
2016	0.090	1442	0.104	1666	0.092	1474
2017	0.097	1610	0.114	1892	0.106	1759
2018	0.095	1562	0.116	1907	0.098	1611
2019	0.095	1582	0.125	2081	0.098	1632
Average	0.104	1584	0.127	1928	0.111	1692
Min	0.090	1378	0.104	1596	0.092	1474
Max	0.124	1876	0.162	2316	0.133	2012

**Fig. 2:**
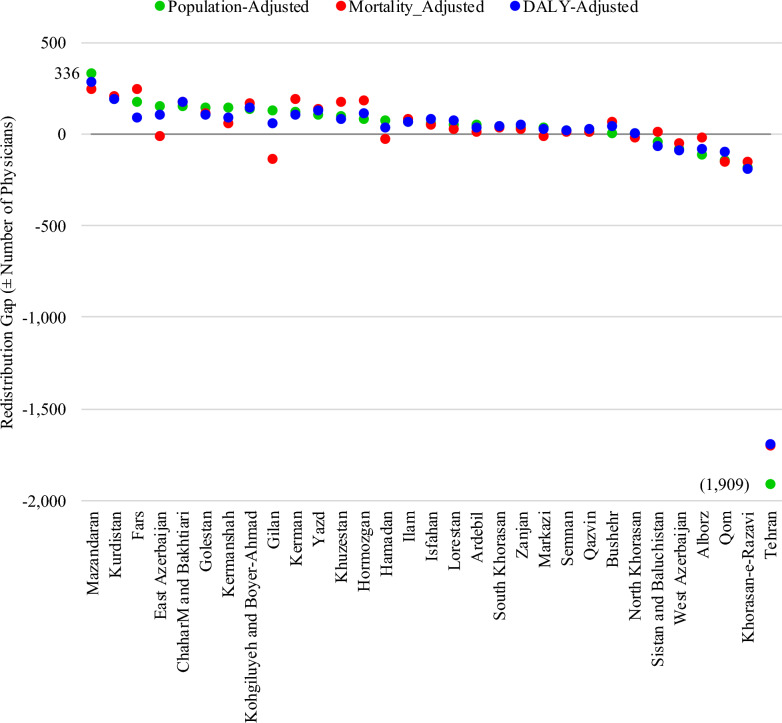
Visualizes the province-level redistribution needs for GPs in 2019

Table 2 presents the number of GPs that needed to be redistributed across Iran during the study period, while Fig. 2 illustrates this data for each province in 2019. According to the results, Tehran (the capital of Iran) exhibited the greatest disparity in GP distribution relative to both its population size and DALY.

**Fig. 3:**
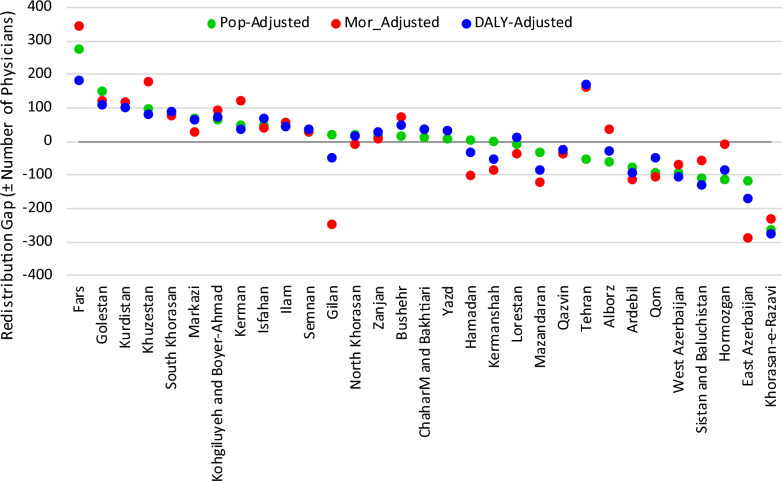
The number of SPs needed to be redistributed in every province of Iran

The trend in the Robin Hood index and the number of SPs requiring redistribution is presented in Table 3. The Robin Hood index for SPs ranged from 0.07 to 0.18 over the study period, reflecting moderate variation in equity across years. On average, the Robin Hood index based on population size for SPs during the study period was 0.118, and it was 0.133 and 0.124 after adjustment for mortality and DALYs, respectively. Overall, the largest redistribution of SPs was required when considering mortality, with 13% of all SPs needing to be reallocated. Based on the results, before and after adjusting for health needs, SP distribution was nearly equitable in 2019, whereas it was notably inequitable in 2006, with the highest redistribution demands driven by mortality. Table 3 presents the number of SPs that needed to be redistributed across Iran during the study period, while Fig. 3 illustrates this data for each province in 2019. According to the results, Tehran exhibited the lowest disparity in SP distribution relative to both mortality and DALY.

**Table 3:** Robin Hood index and redistribution estimates for SPs across provinces (2006–2019)

Year	Based on population		Based on mortality		Based on DALYs	
	Robin Hood index	Physicians to be reallocated(*N*)	Robin Hood index	Physicians to be reallocated(*N*)	Robin Hood index	Physicians to be reallocated(*N*)
2006	0.167	2112	0.189	2390	0.173	2187
2007	0.120	1656	0.148	2042	0.122	1684
2008	0.122	1743	0.138	1972	0.120	1715
2009	0.146	2252	0.115	1774	0.147	2267
2010	0.120	1717	0.135	1932	0.122	1746
2011	0.116	2200	0.129	2447	0.121	2295
2012	0.122	2101	0.127	2187	0.130	2239
2013	0.113	2040	0.124	2238	0.124	2238
2014	0.129	1995	0.152	2351	0.142	2196
2015	0.106	1770	0.113	1887	0.111	1853
2016	0.115	2014	0.130	2276	0.116	2031
2017	0.108	1851	0.130	2228	0.117	2005
2018	0.096	1602	0.123	2053	0.103	1719
2019	0.078	1322	0.104	1763	0.084	1424
Average	0.118	1884	0.133	2110	0.124	1971
Min	0.078	1322	0.104	1763	0.084	1424
Max	0.167	2252	0.189	2447	0.173	2295

**Fig. 4:**
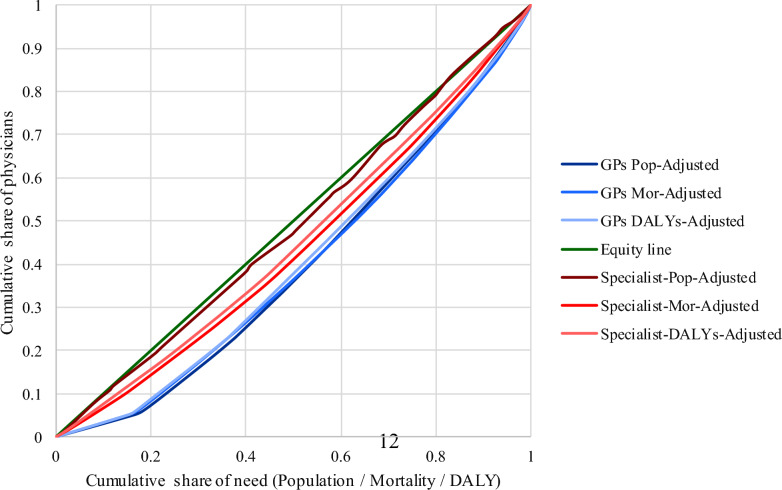
Lorenz curve for GPs and SPs distribution in Iran before and after adjusting health needs

The Lorenz curves in Fig. 4 show that the distribution of both GPs and SPs was relatively close to the line of equality. However, SPs were more evenly distributed than GPs across all health-need scenarios. Among the three need indicators, population size yielded the most equitable results.

## Discussion

A well-performing health workforce is one of the six building blocks of a health system, and equitable distribution is crucial for improving health outcomes and achieving universal health coverage [31]. This study aimed to evaluate the equity in physician distribution by adjusting for population health needs using two key indicators: crude mortality and DALYs. While crude mortality rates slightly declined over the study period, population-adjusted DALYs showed a mild upward trend suggesting a persistent burden of disease, particularly due to chronic and non-communicable conditions. Our findings indicate that physician allocation did not systematically align with these health needs. Contrary to a common assumption that large or wealthier provinces always have a physician surplus, the redistribution analysis revealed that even some populous and resource-rich provinces—such as Tehran—faced significant shortages when physician supply was assessed relative to need. This highlights that physician concentration is not solely a function of province size or wealth, but rather the result of imbalanced policies that overlook need-based planning.

Overall, less than 13% of GPs and SPs would need to be redistributed nationwide to achieve full equity under the adjusted models. However, this seemingly small figure masks deeper imbalances at the subnational level. These findings underscore the importance of shifting from population-based allocation models toward frameworks that prioritize unmet health needs, informed by burden of disease metrics. The findings of this study are consistent with prior research in Iran and other countries that have highlighted inequities in physician distribution when health needs are taken into account. For example, Meshkani et al. [32] and Goudarzi et al. [20], both evaluated physician allocation using mortality and birth rates as proxies for need and concluded that distribution was largely population-based rather than needs-based—mirroring the patterns observed in our own analysis. Similar disparities have been reported internationally. In Albania, Theodorakis et al. [33] applied a range of inequality indices—including Gini and Atkinson—before and after adjusting for mortality and service utilization. Their study demonstrated that failing to account for health needs distorted the perceived fairness of distribution. Yu et al. [34]. analyzing physician equity in China, found that equity appeared acceptable under population-based models but significantly worsened when adjustments were made for service demand and geography—paralleling our results. Other studies have extended this discussion to examine unmet health needs. For instance, Couba et al. used concentration indices to quantify access gaps in Brazil, revealing deep socioeconomic-related inequities. These findings reinforce the importance of considering multiple dimensions—such as income, education, and insurance coverage—when assessing healthcare equity [35].

Disaggregated analysis of redistribution needs for GPs and SPs revealed clear geographic contrasts. Tehran, despite its population and central role in healthcare delivery, showed a notable shortage of GPs relative to health needs, while no such gap was observed for specialists. This may reflect longstanding policies that have focused on rural and underserved areas, potentially neglecting large urban centers, such as Tehran, in GP allocation. In contrast, Fars and Mazandaran, which served as pilot provinces for Iran’s urban family physician program launched in 2012 [36], showed surplus levels of GPs. The higher GP density in these regions likely stems from targeted efforts during the pilot phase. However, the program has seen limited expansion elsewhere. Mohammadibakhsh et al. (2024) highlight how stakeholder resistance, complex payment mechanisms, stewardship and structural issues, financial constraints, cultural elements, and weak referral systems have stalled implementation in other urban areas [37]].

As noted in a qualitative study, the role of GPs remains underdeveloped in Iran’s health system—particularly in large urban centers. The lack of a widespread family medicine and referral structure has led to a confusing patient pathway, where even minor conditions are frequently managed by SPs rather than GPs [38]. These findings underscore the importance of considering not only the numerical distribution, but also the policy environment and system design that shape how physicians are integrated into care delivery.

According to the findings of Mollahaliloglu et al. who analyzed the Gini index of health human resources in Turkey from 2002 to 2016, the country experienced both an overall shortage and significant geographical imbalances in workforce distribution. To address these challenges and promote equity, the authors recommend strategies such as enhancing existing implementation mechanisms, introducing targeted motivation and satisfaction policies, and adopting more comprehensive national planning approaches [39]. A growing body of international research indicated that promoting equitable physician distribution requires a mix of well-aligned strategies. Thailand’s experience offers a compelling example; mandatory rural service, recruiting students from underserved areas, and delivering medical education in non-urban settings have collectively improved workforce equity over time [40]. Global reviews suggest that while financial incentives and compulsory service can ease short-term gaps, lasting impact depends on structural reforms in medical education such as who is trained, where they are trained, and how they are supported [41]. Evidence from European countries also highlights the effectiveness of region-based physician quotas and capitation payment models in aligning physician supply with population needs [42]. A systematic review of global and national experiences confirms the effectiveness of recruiting medical students from underserved regions, integrating rural content in training curricula, and providing incentive packages tied to service in deprived areas [43].

Over the past three decades, Iran has implemented a range of strategies to address the shortage and uneven distribution of physicians in underserved areas. One of the earliest and most enduring approaches has been mandatory service for newly graduated doctors in rural or deprived regions. This policy dates back to the late 1980s and remains in effect to this day [44]. In 2014, a more structured initiative was introduced as part of the Health System Transformation Plan. This program offered enhanced financial packages, including both fixed salaries and performance-based incentives, to physicians working in over 300 designated underserved regions. The goal was not just to deploy physicians, but to ensure their long-term retention through improved compensation and working conditions [45]. Alongside financial and regulatory measures, educational policies have also aimed to promote more equitable distribution. Efforts such as admitting medical students from rural communities, relocating parts of medical training to smaller towns, and providing hands-on clinical experience in underserved areas have all aimed to strengthen ties between future physicians and the communities most in need [46]. The underlying idea is simple but powerful: doctors who feel a connection, whether personal or professional, to a region are more likely to stay and serve there. These initiatives reflect a shift in thinking. It is no longer just about filling positions, but about investing in people, building local capacity, and creating meaningful, long-term career opportunities. Still, despite these positive steps, major challenges remain in building sustainable systems that truly support health workers where they are needed most. Systematic reviews of Iran’s family physician program reveal structural weaknesses in the referral system, inconsistent gatekeeping functions, and inadequate planning for deploying GPs effectively especially in urban or underserved regions [46]. Additionally, educational barriers persist. Reviews highlight that inadequate student selection policies, insufficient rural exposure during training, and outdated curricula deter newly graduated GPs from serving in deprived areas [47].

This study highlighted that, while the distribution of GPs and SPs in Iran has improved over time, significant disparities remain, particularly when adjusted for actual health needs. This study offered different insights into how physicians, both general practitioners and specialists, are distributed across Iran. Although the overall pattern suggests some progress toward equity, important gaps remain, especially when we look beyond population numbers and consider actual health needs like mortality and disease burden. Our findings show that using population size alone as a planning tool can be misleading. Instead, health workforce policies should take a broader view—one that includes how people use services, how demographics are changing, and how local socioeconomic conditions shape health needs. Tools like telemedicine, financial incentives, and stronger career development plans may also help encourage doctors to stay longer in underserved areas.

That said, the study is not without limitations. We did not account for variations within provinces—for example, differences between urban and rural areas. We also did not explore other factors that could affect where doctors work, like infrastructure readiness or local health policies. And while DALYs and mortality rates help quantify health needs, they do not necessarily reflect patterns of care-seeking or changes in how diseases are managed over time. In short, if the goal is a more balanced and fair distribution of healthcare workers, especially in high-need areas, a one-size-fits-all approach will not work. Policymakers will need to rely on a mix of data, long-term planning, and policies that go beyond numbers, and instead look closely at what people need.

## Conclusion

Policymakers should have a trade-off between health needs and resource allocation. Allocating resources without considering people's health needs will not improve health outcomes. Focus on the health needs of a community, including the age and gender composition, socioeconomic variables, burden of disease, incidence, and prevalence of diseases, pandemics, and birth rate could help policymakers reach equity. Other indicators can also be identified and defined according to the health resource allocation. While Iran’s physician numbers have climbed, raw growth has not translated into equitable, needs-adjusted distribution. A substantial share of both GPs and SPs remains clustered in lower-need provinces, leaving high-burden regions underserved. To bridge this gap, policymakers must deploy targeted incentives, enforce redistribution mechanisms, and embrace innovative service models like telehealth.

## Data Availability

No datasets were generated or analysed during the current study.

## R commands to calculate the Robin Hood index

Data <—read.csv("C:/Users/user /data.csv").

head(data).

install.packages("dplyr").

library(dplyr).

data <—data % > % mutate(gp_per_capita = gp/pop).

data <—data % > % mutate(prop_gp = gp_per_capita/sum(gp_per_capita, na.rm = TRUE)).

data <—data % > % arrange(prop_gp) % > % mutate(cum_prop = cumsum(prop_gp)).

n <—nrow(data).

equal_dist <—seq(1/n, 1, length.out = n).

robin_hood_index <—max(abs(data$cum_prop—equal_dist)).

print(paste("Robin Hood Index (H):", round(robin_hood_index, 3))).

## Abbreviations

GPs: General practitioners
SPs: Specialists
DALY: Disability-adjusted life years
GBD: Global burden of disease
WHO: World Health Organization
UHC: Universal health coverage
HRH: Human resources for health
HNI: Health needs index
